# Characterization of 3-Oxacyl-Acyl Carrier Protein Reductase Homolog Genes in *Pseudomonas aeruginosa* PAO1

**DOI:** 10.3389/fmicb.2019.01028

**Published:** 2019-05-22

**Authors:** Qiao-Qiao Guo, Wen-Bin Zhang, Chao Zhang, Yu-Lu Song, Yu-Ling Liao, Jin-Cheng Ma, Yong-Hong Yu, Hai-Hong Wang

**Affiliations:** ^1^Guangdong Provincial Key Laboratory of Protein Function and Regulation in Agricultural Organisms, College of Life Sciences, South China Agricultural University, Guangzhou, China; ^2^Guangdong Food and Drug Vocational College, Guangzhou, China

**Keywords:** *Pseudomonas aeruginosa*, 3-oxoacyl-ACP reductase, fatty acid synthesis, quorum-sensing, pathogenesis

## Abstract

Bacterial 3-oxoacyl-ACP reductase (OAR) catalyzes the 3-oxoacyl-ACP reduction step in the fatty acid synthesis pathway. At least 12 genes in the *Pseudomonas aeruginosa* genome are annotated as OAR-encoding genes. In this study, we characterized the functions of these genes with biochemical and genetic techniques. With the exception of PA2967, which encodes FabG, an essential protein in fatty acid synthesis, only the PA4389 and PA4786 gene products had OAR activity, and the single deletion of these two genes reduced the ability of *P. aeruginosa* to produce several specific quorum-sensing (QS) signals. However, PA4389 and PA4786 do not have key roles in fatty acid synthesis. Moreover, although most OAR homologs had no OAR activity, some may function in carbon utilization. The PA3128 product may play a role in the TCA cycle, and PA0182 and PA1470 seem to be required for the utilization of several amino acids. The rest of the OAR homologs have no roles in carbon utilization, but the deletion of one of these genes might affect the production of virulence factors by *P. aeruginosa*. We conclude that most OAR homolog genes do not encode OAR enzymes, and that these proteins do not function in fatty acid synthesis.

**Importance:**

We report that although all *P. aeruginosa* OAR homologs have similar structures and the conserved catalytic triad of the bacterial OAR enzymes, only a few OAR homologs have OAR activity.

## Introduction

Bacterial 3-oxoacyl-ACP reductase (OAR), also called FabG, catalyzes the 3-oxoacyl-ACP reduction step in the fatty acid synthesis pathway (Campbell and Cronan, 2001; Lu et al., 2004; White et al., 2004; Zhang and Rock, 2008). The *fabG* gene is usually located in the bacterial fatty acid synthetic gene cluster, which is ubiquitously expressed and highly conserved across bacterial species (Campbell and Cronan, 2001; Price et al., 2001). FabG is thought to be the only protein isoform that functions as a 3-oxoacyl-ACP reductase in the bacterial fatty acid synthesis pathway (Price et al., 2001; White et al., 2004). However, exceptions to this have been shown to exist in several bacteria. *Ralstonia solanacearum*, a soil-borne plant pathogen, has two functional FabGs, although only FabG1 is required for bacterial growth (Feng et al., 2015). In *Sinorhizobium meliloti*, *nodG*, in the *nodFEG* operon, also encodes an OAR, which can replace the function of FabG in fatty acid synthesis when it is overexpressed (Lopez-Lara and Geiger, 2001; Mao et al., 2016). XCC0416 in *Xanthomonas campestris* pv. *campestris* encodes a novel OAR that specifically reduces long-chain 3-oxoacyl-ACP to 3-hydroxyacyl-ACP, and it can function in *X. campestris* pv. *campestris* fatty acid synthesis when it is expressed at sufficiently high levels and the medium is supplemented with octanoic acid (Hu et al., 2018). These findings suggest that one bacterial species can simultaneously express two or more OAR isozymes, like other fatty acid synthesis enzymes.

Bacterial OAR is a member of a very large family of enzymes, the short-chain alcohol dehydrogenase/reductase (SDR) family, which catalyzes a wide range of oxidation–reduction reactions in many metabolic pathways, using NADH or NADPH as the cofactor (Kallberg et al., 2002; Oppermann et al., 2003). One bacterial species generally encodes many SDR family proteins (Lai and Cronan, 2004; Wang and Cronan, 2004), but only a few SDR proteins have been shown to have OAR activity. For example, the *S. meliloti* genome contains 77 genes that are categorized within the SDR family (Jacob et al., 2008), but to date, only *fabG* and *nodG* have been shown to encode proteins with OAR activity (Lopez-Lara and Geiger, 2001; Mao et al., 2016). Therefore, it can be very difficult to distinguish a true OAR from other SDR family members that catalyze unrelated reactions with a bioinformatics analysis.

*Pseudomonas aeruginosa* is an aerobic Gram-negative bacterium that lives in soil and aqueous environments (Driscoll et al., 2007; Lee and Zhang, 2015). It is also an important opportunistic pathogen in humans, responsible for a myriad of infections, including serious bacteremia and nosocomial pneumonia, and it is the major cause of morbidity and mortality among cystic fibrosis patients (Kerr and Snelling, 2009; Moore and Flaws, 2011). Its intrinsic resistance to most conventional antibiotics and the formation of a biofilm matrix during infection make *P. aeruginosa*-related infections hard to eradicate (Lee and Zhang, 2015), hence the development of new anti-pseudomonas drugs is a challenging priority.

In *P. aeruginosa*, the fatty acid biosynthetic pathway plays a multifaceted role in maintaining both its viability and virulence (Hoang et al., 2002; Zhu and Rock, 2008; Yuan et al., 2012a). A wide range of cellular processes are dependent on this pathway, ranging from the biosynthesis of essential cellular structural components and cofactors to the production of diffusible secondary metabolites, such as the quorum-sensing (QS) signal molecules, rhamnolipids and siderophore pyoverdine (Raetz and Whitfield, 2002; Zhu and Rock, 2008; Spalding and Prigge, 2010; Hannauer et al., 2012; Jimenez et al., 2012). Therefore, the fatty acid biosynthetic pathway in *P. aeruginosa* is considered an attractive target for the development of new anti-pseudomonas agents. However, fatty acid synthesis is more complex in *P. aeruginosa* than in *Escherichia coli*. Although the basic steps in the fatty acid synthetic pathway of *P. aeruginosa* are similar to those in *E. coli* (Hoang et al., 2002), at least three *P. aeruginosa* enzymes do not occur in the fatty acid synthetic pathway of *E. coli*. First, unlike *E. coli* and several other bacteria, *P. aeruginosa* lacks a FabH homolog, but uses FabY, a newly identified 3-ketoacyl synthase KAS I/II domain enzyme, to condense acetyl coenzyme A with malonyl-ACP to initiate *de novo* fatty acid synthesis (Yuan et al., 2012b). Second, *P. aeruginosa* uses PA3286, which condenses octanoyl-CoA with malonyl-ACP, to produce the intermediate 3-ketodecanoyl-ACP, and shunts β-oxidation degradation intermediates into *de novo* fatty acid synthesis (Yuan et al., 2012a). Third, as well as FabI, *P. aeruginosa* has a second enoyl-ACP reductase, FabV, which makes *P. aeruginosa* highly resistant to triclosan (Zhu et al., 2010; Huang et al., 2016).

The *P. aeruginosa* genome encodes 42 SDR family proteins, 12 of which are annotated as OAR homologs (Stover et al., 2000). However, the physiological functions of these SDR proteins, especially the OAR homologs, are poorly understood. To date, only two OAR homologs have been studied. *fabG* (PA2967), located in the *fabD*-*fabG*-*acpP*-*fabF* gene cluster, is an essential gene in the fatty acid synthetic pathway of *P. aeruginosa* (Kutchma et al., 1999; Hoang et al., 2002). RhlG, encoded by PA3387, the other OAR homolog, was first identified as a 3-ketoacyl reductase specifically involved in rhamnolipid synthesis (Campos-Garcia et al., 1998). However, structural and genetic studies have shown that RhlG does not catalyze the reduction of 3-oxodecanoyl-ACP to 3-hydroxydecanoyl-ACP *in vitro* and is not involved in rhamnolipid production *in vivo* (Miller et al., 2006; Zhu and Rock, 2008).

In this study, we investigated the functions of 12 OAR homologs of *P. aeruginosa* in fatty acid synthesis, carbohydrate metabolism, the production of QS signals, and pathogenesis using genetic and biochemical techniques. In this way, we propose that the *P. aeruginosa* OAR homologs do not function in fatty acid synthesis.

## Materials and Methods

### Bacterial Strains, Plasmids, and Growth Media

The *E. coli* K-12 strain, the *P. aeruginosa* PAO1 strain, and the plasmids used in this study are listed in Supplementary Table S1. Luria-Bertani (LB) broth was used as the rich medium for *E. coli* or *P. aeruginosa* growth at 37°C. The *E. coli fabG* (Ts) mutant strain CL104 (Kutchma et al., 1999) was grown in RB medium (10 g/l tryptone, 10 g/l NaCl, and 1 g/l yeast extract) at 30°C. ME medium (Zhu et al., 2010) supplemented with 0.1% yeast extract was used to screen the *P. aeruginosa* mutants, and if needed, 10% sucrose was added. Antibiotics were added at the following concentrations (in μg/ml): sodium ampicillin, 100; kanamycin sulfate, 30; and gentamicin sulfate, 30. L-Arabinose was used at a final concentration of 0.01%. Isopropyl-β-D-thiogalactoside (IPTG) was used at a final concentration of 1 mM, and 5-bromo-4-chloro-3-indolyl-β-D-galactoside (*X*-Gal) at a final concentration of 20 μg/ml. Bacterial growth in liquid medium was determined by measuring the optical density at 600 nm (OD_600_) with the Bioscreen C Automated Microbiology Growth Curve Analysis System (OY Growth Curves).

The carbon utilization phenotypes of the *P. aeruginosa* mutants were screened on Biolog Gen III MicroPlates^TM^, with the 68 carbon sources listed in Supplementary Table S4. The assays were performed according to the instructions provided by the manufacturer. After incubation at 37°C for 12 h, an OD_600_ > 0.1 for wells containing a mutant strain indicated that the strain utilized that specific carbon source. Three independent experiments were performed for each mutant strain.

### Recombinant DNA Techniques and Construction of Plasmids

Twelve DNA fragments containing putative OAR genes were amplified from the *P. aeruginosa* PAO1 genomic DNA with *Pfu* DNA polymerase and the specific primers listed in Supplementary Table S2. The products were inserted into the pMD19-T vector to generate 12 pMD19-derived plasmids, which are listed in Supplementary Table S1. The foreign DNA fragments in these T-vectors were digested with *Nde*I and *Hin*dIII or *Sal*I. The appropriate fragments were isolated and ligated into pBAD24M (Zhu et al., 2010) and pET-28 (b) to create 12 pBAD24M-derived plasmids and 12 pET28b-derived plasmids, respectively. All these plasmids are listed in Supplementary Table S1. Using the same process, we also constructed the *E. coli fabG*-encoding plasmids pMD19-EcfabG and pET28b-EcfabG.

### Disruption of the OAR Genes

To construct the gene knockout mutants, a *sacB*-based strategy was used, as described previously (Mao et al., 2016). Using *P. aeruginosa* genomic DNA as the template, the 600-bp DNA fragments upstream (Up fragment) and downstream (Down fragment) from a specific OAR gene were amplified with *Pfu* DNA polymerase and the specific primers are shown in Supplementary Table S2. The PCR products were purified and an overlap extension PCR was used to fuse the Up and Down fragments of the specific OAR genes. The 1200-bp knockout cassette fragment was then inserted into plasmid pK18mobsacB (Schafer et al., 1994) to create a suicide plasmid, which was used to knock out the specific OAR gene. We constructed 12 suicide plasmids, which are listed in Supplementary Table S1. All these plasmids were introduced into *P. aeruginosa* PAO1 with conjugal transfer from *E. coli* S17-1. The OAR mutant strains were screened as described previously (Mao et al., 2016). Eleven OAR-gene-deleted strains were generated and are listed in Supplementary Table S1. However, we could not create a PA2967-deleted strain.

### Purification of OAR Proteins and Assay of OAR Activity *in vitro*

The pET28b-derived plasmids that carried the 12 OAR encoded genes were introduced into *E. coli* BL21 (DE3) cells and the 12 OAR proteins were strongly expressed (data not shown). The 12 OAR proteins were then purified with nickel chelate chromatography, as described previously (Mao et al., 2016). *E. coli* FabD, FabH, FabG, FabZ, and FabI, *Vibrio harveyi* acyl-ACP synthetase (AasS), *R. solanacearum* RSp0194 protein (Mao et al., 2015), and *E. coli* holo-ACP were also purified as described previously (Zhu et al., 2010). The OAR activities were tested *in vitro*, as described previously (Mao et al., 2016). Briefly, malonyl-ACP was synthesized from holo-ACP and malonyl-CoA using *E. coli* FabD, and octanoyl-ACP was synthesized from octanoic acid, ATP, and holo-ACP using *V. harveyi* AasS, as described previously (Zhu et al., 2010). The ability of an OAR enzyme to reduce 3-oxobutyryl-ACP to 3-hydroxybutyryl-ACP during the initiation reaction of the fatty acid synthesis cycle was assessed in reaction mixtures containing 0.1 M sodium phosphate (pH 7.0), 0.1 μg each of *E. coli* FabH, FabZ, and FabI, 50 μM NADH, 50 μM NADPH, 1 mM β-mercaptoethanol, 100 μM acetyl-CoA, 100 μM malonyl-CoA, and 50 μM holo-ACP in a final volume of 40 μl. The reaction mixtures used to investigate the reduction of long-chain 3-ketoacyl-ACP contained 0.1 M sodium phosphate (pH 7.0), 50 μM malonyl-ACP, 50 μM octanoyl-ACP, 0.1 μg each of *R. solanacearum* RSp0194 protein, *E. coli* FabZ, and *E. coli* FabI, 50 μM NADH, and 50 μM NADPH. The reactions were initiated by the addition of 0.1 μg of OAR enzyme and incubated for 1 h. The reaction products were resolved with conformation-sensitive gel electrophoresis on 20% polyacrylamide gel containing a concentration of urea optimized for the separation. The gel was stained with Coomassie Brilliant Blue R250.

### Analysis of the Fatty Acid Compositions of OAR Strains

Cultures were grown aerobically at 37°C in LB medium for 12 h. The cells were then harvested from 10 ml of culture and washed with fresh LB medium at room temperature. Fatty acid methyl esters were extracted with a previously reported procedure (Zhu et al., 2010), and the fatty acid methyl esters of each strain were analyzed with gas chromatography–mass spectrometry (GC–MS), as previously described (Basconcillo and McCarry, 2008). The data are presented as percentages of the total fatty acids and represent the means ± standard errors of the means of three independent determinations.

### Extraction and Assay of QS Signal Molecules

The QS signal molecules were extracted as described previously (Yuan et al., 2012b). *P. aeruginosa* cells were grown in LB broth to early stationary phase, and were then removed from 10 ml of growth medium by centrifugation at 12,000 × g for 15 min at 4°C. The QS signal molecules were extracted twice from 10 ml of supernatant from each sample, using either an equal volume of ethyl acetate for the homoserine lactones (HSL) or an equal volume of acidified ethyl acetate for 2-heptyl-3-hydroxy-4-quinolone (PQS). The organic extracts were concentrated to dryness with a nitrogen bubbler, and the residues were resuspended in 100 μl of ethyl acetate (HSL) or methanol (PQS). To detect AHL-like molecules with short acyl chains, the biosensor *Chromobacterium violaceum* CV026 was used (McClean et al., 1997). *Agrobacterium tumefaciens* NTL4 (pZLR4) was used to detect AHLs with long acyl chains (Cha et al., 1998). The PQS content was determined with normal-phase TLC on activated silica 60 F254 plates (Merck), according to a previously described method (Yuan et al., 2012b). Authentic PQS (Sigma) was used as the standard. At least three independent repetitions of each experiment were conducted to calculate the parameters.

**Table 1:** Alignment of *P. aeruginosa* OAR homologs with *E. coli* FabG.

OAR homologs	Length	Identities (%)	N-terminal cofactor binding motif (Gly12-ASR-Gly16-I-Gly18)	Catalytic motif (Ser138, Tyr151-AAA-Lys155)	Pfam analysis (Motif ID)
PA0182	251	35.6	Gly17-GSR-Gly21-I-Gly23	Ser145, Tyr159-AMS-Lys163	adh_short; KR; Epimerase; adh_short_C2
PA1470	245	33.7	Gly12-ASR-Gly16-I-Gly18	Thr140, Tyr153-IAS-Lys157	adh_short; KR; Epimerase; adh_short_C2
PA1827	253	39.9	Gly15-GSS-Gly19-I-Gly21	Ser142, Tyr157-AAA-Lys161	adh_short; KR; Epimerase; adh_short_C2
PA2142	286	33.7	Gly49-GDS-Gly53-I-Gly55	Ser178, Tyr191-SST-Lys195	adh_short; KR; Epimerase; adh_short_C2
PA2967	247	63.6	Gly12-ASR-Gly16-I-Gly18	Ser141, Tyr154-AAA-Lys158	adh_short; KR; Epimerase; adh_short_C2
PA3128	248	32.7	Gly9-ASR-Gly13-I-Gly15	Ser143, Tyr157-AAA-Lys161	adh_short; KR; Epimerase; adh_short_C2
PA3387	256	32.9	Gly16-GSR-Gly20-I-Gly22	Ser148, Tyr162-GPS-Lys166	adh_short; KR; Epimerase; adh_short_C2
PA4089	253	37.9	Gly13-AGQ-Gly17-I-Gly19	Ser142, Tyr156-AAS-Lys160	adh_short; KR; Epimerase; adh_short_C2
PA4389	252	34.7	Gly12-GCQ-Gly16-L-Gly18	Ser149, Tyr162-SAA-Lys166	adh_short; KR; adh_short_C2
PA4786	451	37.9	Gly221-AAR-Gly225-I-Gly227	Ser344, Tyr357-AVS-Lys361	adh_short; KR; Epimerase; adh_short_C2
PA5150	245	29.3	Gly11-ASR-Gly15-I-Gly17	Ser142, Tyr156-ATS-Lys160	adh_short; Epimerase; adh_short_C2
PA5524	260	38.7	Gly16-AGSEL-Gly22-I-Gly24	Ser152, Tyr166-SAA-Lys170	adh_short; KR; adh_short_C2

### Biofilm Mass Evaluation

A crystal violet staining protocol (Hinsa and O’Toole, 2006) was used to quantify the biofilm mass. Briefly, after the bacterial cells were incubated, the culture medium was carefully pipetted from the individual wells of a 96-well plate, and the plate was gently washed three times with PBS to remove any loosely attached cells. The remaining biofilms attached to the wells were then fixed with 150 μl of absolute methanol for 15 min and air-dried at room temperature for 10 min. Next, 100 μl of 1% (w/v) crystal violet was added and allowed to stain the samples for 20 min. Excess crystal violet was removed by rinsing the wells three times with sterile distilled water. The crystal-violet-stained material was then dissolved in absolute ethanol, and the amount of stained biomass was measured indirectly with spectrometry (optical density at 595 nm). At least three independent repetitions were performed when measuring the biofilm mass.

### Motility Assay

The motility assay was performed as described previously (Huang et al., 2016). The swarming motilities of the *P. aeruginosa* strains were investigated on medium containing 0.45% tryptone, 0.13% yeast extract, 0.22% NaCl, 0.5% glucose, and 0.5% agar. The agar medium was air-dried for 15 min before use. For the swarming assays, the surfaces of the plates were point-inoculated with bacteria from an overnight culture using a sterile toothpick, and then incubated at 30°C for 24–48 h.

### Assay of Other Virulence Factors

A siderophore secretion assay of the *P. aeruginosa* strains was performed on chrome azurol S (CAS)–LB agar plates, as described previously (Huang et al., 2016; Ma et al., 2017). The quantitation of the pyocyanin produced by the *P. aeruginosa* strains was based on the absorbance of pyocyanin in acidic solution at a wavelength of 520 nm, and the pyocyanin was extracted with chloroform and 0.2 mmol/l HCl. The LasB protease activity in the *P. aeruginosa* culture supernatants was measured with the elastin Congo red assay (Yuan et al., 2012b), and the LasA protease activity was determined by measuring the ability of the *P. aeruginosa* culture supernatants to lyse boiled *Staphylococcus aureus* RN4220 cells, as described previously (Huang et al., 2016). At least three independent repetitions of each experiment were performed to calculate the parameters.

### Statistical Analysis

All data were analyzed with analysis of variance (ANOVA) using the JMP software, version 5.0 (SAS Institute Inc., Cary, NC, United States). Pairwise comparisons were then made between all groups with Tukey’s method. Significant effects of treatment were determined with *F*-values. *P* < 0.05 was regarded as significant. All measurements were made in triplicate.

## Results

### Bioinformatic Analysis of OAR Homologs of *P. aeruginosa*

To identify novel 3-oxoacyl-ACP reductases in *P. aeruginosa*, a BLAST analysis of the *P. aeruginosa* PAO1 genome (Stover et al., 2000) was performed using *P. aeruginosa* FabG as the query sequence. Of the 42 SDR encoded in the *P. aeruginosa* genome, nine are putative OARs: PA0182, PA1470, PA1827, PA2967 (FabG), PA3387 (RhlG), PA4089, PA4389, PA4786, and PA5524. We also included PA2142, PA3128, and PA5150 in this study because their putative proteins share high identities with *P. aeruginosa* FabG and were among the top 10 of these 42 SDR proteins most similar to *P. aeruginosa* FabG in an amino acid sequence alignment.

*Escherichia coli* FabG is a typical bacterial OAR, the biological functions and crystal structure of which have been studied extensively (Price et al., 2001; Lai and Cronan, 2004). Therefore, a multiple-sequence alignment of *E. coli* FabG and the *P. pseudomonas* OAR homologs was constructed with ClustalW (Table 1). The alignment showed that *P. aeruginosa* FabG shares only 63.6% identity with *E. coli* FabG, whereas the remaining OAR homologs share amino acid sequence identities of around 29.3–39.9% with *E. coli* FabG. However, the N-terminal cofactor-binding motif (Gly–X3–Gly–Ile–Gly) present in *E. coli* FabG is conserved in all OAR homologs, except PA5524, in which the motif is Gly–X5–Gly–Ile–Gly (Table 1). The catalytic triad (Ser–Tyr–X3–Lys) of the SDR proteins is also present in these OAR homologs, except PA1470, in which the Thr–Tyr–X3–Lys triad replaces the Ser–Tyr–X3–Lys triad (Table 1). A Pfam analysis in KEGG showed that all these OAR homologs, except PA4389, contain the adh_short, KR, and adh_short_C2 motifs, among others. The epimerase motif is also present in these OAR homologs (Table 1).

At the website http://swissmodel.expasy.org/interactive, we used Swiss-Modeling to simulate protein structural models for 11 OAR homologs (Supplementary Table S3). The structural models of six OAR homologs, PA1827, PA3387, PA4089, PA4389, PA5150, and PA5524, were based on *E. coli* FabG (PDB no. 1q7c) as the template. The models for PA1470 and PA3128 were built based on *S. aureus* FabG1 (PDB no. 3sj7) as the template. The protein structural models for PA0182, PA2142, and PA4786 were simulated with *P. aeruginosa* FabG (PDB no. 4bny), *Synechocystis* sp. FabG (PDB no. 4rzh), and *Mycobacterium tuberculosis* FabG4 (3v1t) as templates, respectively. The quality of all these structural models was high, their GMQE values were around 0.63–0.73, and their QMEAN values ranged from −0.22 to −3.49 (Supplementary Table S3). Based on these criteria, it is reasonable to infer that all these OAR homologs have OAR activity.

### Not All OAR Homologs Have OAR Activity

**FIGURE 1:**
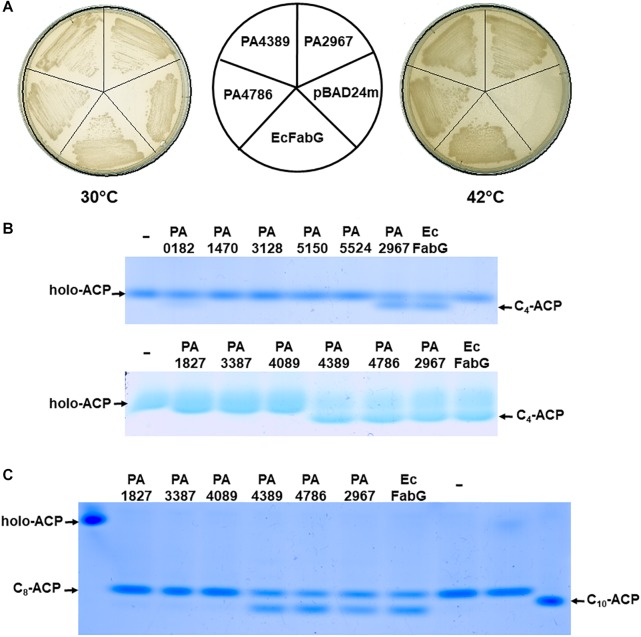
Examination of OAR activities of *P. aeruginosa* OAR homolog proteins *in vivo* or *in vitro*. **(A)** Complementation of the *E. coli fabG*(Ts) strain CL104 by expression of *P. aeruginosa* OAR homolog proteins. The *E. coli* FabG (EcFabG), PA2967, PA4389 and PA4786 proteins were expressed from pBAD24M-derived plasmids listed in Supplementary Table S1. **(B)** *P. aeruginosa* OAR homolog proteins function in the first cycle of fatty acid biosynthesis. **(C)** OAR homolog proteins function in the elongation reactions of fatty acid biosynthesis. C_4_-ACP, butyryl-ACP; C_8_-ACP, octanoyl-ACP; C_10_-ACP, decanoyl-ACP. The pET28b-derived plasmids, which carried 12 OAR encoded genes, were expressed in *E. coli* BL21 (DE3) and OAR proteins were purified by nickel chelate chromatography. The fatty acid biosynthetic reactions were reconstructed *in vitro* as described in Section “Materials and Methods.”

To determine whether 12 *P. aeruginosa* OAR homologs have OAR activity, each of these genes was inserted into the arabinose-inducible vector pBAD24M to generate 12 expression constructs, which are listed in Supplementary Table S1. The plasmids were transferred into the *E. coli fabG* temperature-sensitive (Ts) mutant strain CL104 (Lai and Cronan, 2004) at the permissive temperature (30°C). The resulting transformants were tested for their growth on rich broth (RB) plates at the non-permissive temperature (42°C). As expected, the derivative of CL104 that carried the PA2967 (*fabG*)-encoding plasmid grew well at 42°C in the absence of arabinose (Figure 1A). CL104 carrying either the PA4389- or PA4786-encoding plasmid also grew at 42°C (Figure 1A), indicating that PA4389 and PA4786 have OAR activity *in vivo*. However, the remaining OAR-homolog-encoding genes did not allow *E. coli* CL104 to grow at 42°C, even in the presence of arabinose (data not shown). These data suggest that not all *P. aeruginosa* OAR homologs have OAR activity *in vivo*, even though they were annotated as OAR enzymes in a bioinformatic analysis.

To further test this hypothesis, the enzymatic activities of 12 *P. aeruginosa* OAR homologs were determined *in vitro*. Each OAR-homolog-encoding gene was cloned into the vector pET28 (b) to generate 12 protein expression plasmids, which are listed in Supplementary Table S1. The OAR homolog proteins were then expressed in *E. coli* BL21 (DE3) and the N-terminal-His_6_-tagged versions of the proteins were purified with nickel chelate chromatography (data not shown). To test the relative activities of the OARs, the *E. coli* fatty acid biosynthetic proteins FabD, FabH, FabG, FabZ, FabI, and holo-ACP, *V. harveyi* AasS, and *R. solanacearum* RSp0194 were purified with previously described protocols (Zhu et al., 2010; Mao et al., 2016) (data not shown).

First, the enzymatic activities of the OAR homologs in the initial step of fatty acid synthesis were tested. The initial reaction was reconstituted by the sequential addition of FabD, FabH, OAR, FabZ, and FabI, and was followed by incubation for 1 h at 37°C and analysis of the reaction products with conformationally sensitive gel electrophoresis. In the initial reactions, FabD converts malonyl-CoA to malonyl-ACP, FabH condenses acetyl-CoA with malonyl-ACP to produce 3-oxobutyryl-ACP, OAR catalyzes the reduction of 3-oxobutyryl-ACP to 3-hydroxybutyryl-ACP, FabZ dehydrates 3-hydroxybutyryl-ACP to produce *trans*-2-butenoyl-ACP, and FabI reduces *trans*-2-butenoyl-ACP to butyryl-ACP (Campbell and Cronan, 2001; White et al., 2004). Therefore, if an OAR homolog does not have OAR activity, only holo-ACP is seen on the gel, whereas the addition of an active OAR enzyme to the reaction results in the presence of butyryl-ACP on the gel (Figure 1B). The results showed that the mixtures containing PA2967, PA4389, or PA4786 produced a clear butyryl-ACP band on the gel, indicating that PA2967, PA4389, and PA4786 can reduce 3-oxobutyryl-ACP and function in the initial reaction of fatty acid synthesis. However, the reactions containing the remaining of OAR homologs did not produce a butyryl-ACP band on the gel (Figure 1B), suggesting that these OAR homolog proteins do not play a role in the initial reactions of fatty acid synthesis.

The enzymatic activities of the OAR homologs in the reduction of long-chain 3-ketoacyl-ACP substrates were then examined. The reaction was reconstituted by combining malonyl-ACP, octanoyl-ACP, *R. solanacearum* RSp0194, OAR, FabZ, and FabI. In the reaction, *R. solanacearum* RSp0194 condenses octanoyl-ACP with malonyl-ACP to produce 3-oxodecanoyl-ACP (Mao et al., 2015), OAR converts 3-oxodecanoyl-ACP to 3-hydroxydecanoyl-ACP, FabZ dehydrates 3-hydroxydecanoyl-ACP to *trans*-2-decenoyl-ACP, and FabI reduces *trans*-2-decenoyl-ACP to decanoyl-ACP (Campbell and Cronan, 2001; Wang and Cronan, 2004). With the addition of *E. coli* FabG, the mixture produced a decanoyl-ACP band on the gel, whereas with the addition of no OAR protein, the reaction only produced an octanoyl-ACP band (Figure 1C). The reaction mixtures containing PA2967, PA4389, or PA4786 produced octanoyl-ACP and decanoyl-ACP bands on the gel, as when *E. coli* FabG was added to the reaction (Figure 1C), indicating that PA2967, PA4389, and PA4786 catalyze the reduction of long-chain 3-ketoacyl-ACPs. However, the remaining OAR homologs did not catalyze 3-oxodecanoyl-ACP reduction (Figure 1C). These data confirm that PA2967, PA4389, and PA4786 have OAR activity and function in the fatty acid synthetic reaction *in vitro*. These results are consistent with the ability to support the growth of *E. coli* strain CL104 at the non-permissive temperature, and confirm that not all *P. aeruginosa* OAR homologs have OAR activity, even though they share high structural similarity with some real OAR enzymes.

### OAR Homologs Are Not Essential for *P. aeruginosa*

A phenotypic analysis can be a very powerful way to determine gene functions. Therefore, to understand the role of the 12 OAR homologs in *P. aeruginosa*, each gene was deleted from the genome and the phenotypes of the corresponding mutants were analyzed. To create an unmarked deletion mutant, overlapping PCR was used to fuse the DNA fragments flanking the target gene, and the product was inserted into pK18mobscaB (Schafer et al., 1994) to generate the plasmid used to delete the target gene. The plasmid carrying the in-frame gene deletion was introduced into the genome of wild-type *P. aeruginosa* strain PAO1 by conjugal transfer from *E. coli* S17-1. The mutant strain was isolated with negative selection mediated by *Bacillus subtilis sacB*, and confirmed by sequencing the PCR fragment of the disrupted allele. With this strategy, we obtained 11 OAR homolog mutants, but no PA2967 (*fabG*) mutant (Supplementary Table S1).

Next, we tested the growth of these mutants in rich or minimal medium. All the mutant strains grew as well as the wild-type stain PAO1 in both media (data not shown), indicating that these OAR homologs, except PA2967 (*fabG*), are not essential for *P. aeruginosa*.

**Table 2:** Analysis of fatty acid compositions in *P. aeruginosa* mutant strains^α^.

Fatty acid^β^	Composition (%)				
	PAO1	ΔPA0182	ΔPA3387	ΔPA4389	ΔPA4786
n-C_12:0_	3.11 ± 0.91	3.05 ± 0.65	3.32 ± 0.68	2.14 ± 0.22	2.77 ± 0.27
n-3-OH-C_14:0_	17.69 ± 1.75	20.97 ± 4.87	13.35 ± 5.09	16.07 ± 1.49	14.47 ± 3.19
n-C_14:0_	0.50 ± 0.00	0.67 ± 0.09	0.58 ± 0.06	0.39 ± 0.04	0.44 ± 0.01
n-C_16:1_	8.01 ± 0.46	10.41 ± 1.39	9.49 ± 0.79	5.82 ± 0.54	6.15 ± 0.54
n-C_16:0_	30.52 ± 1.47	28.27 ± 1.64	31.53 ± 1.61	29.45 ± 1.29	31.31 ± 1.05
n-C_18:1_	35.01 ± 1.88	32.39 ± 2.95	36.76 ± 2.19	34.25 ± 1.26	33.66 ± 2.04
n-C_18:0_	3.17 ± 0.40	2.31 ± 0.78	2.54 ± 0.18	3.42 ± 0.47	2.46 ± 0.11
n-C_17:0_-cyc	3.21 ± 0.90	2.12 ± 0.65	2.43 ± 0.70	8.60 ± 1.74	8.74 ± 1.34

*^α^Total lipids were extracted and transesterified to generate fatty acid methyl esters, and the products were identified with GC–MS. Values are the means ± standard deviations of three independent experiments and percentages of total fatty acids. ^β^n-C_12:0_, dodecanoic acid; n-C_14:0_-3-OH, 3-hydroxyl-tetradecanoic acid; n-C_14:0_, tetradecanoic acid; n-C_16:1_, palmitoleic acid; n-C_16:0_, palmitic acid; n-C_18:1_, cis-11-octadecenoic acid; n-C_18:0_, octadecanoic acid; n-C_17:0_-cyc, cis-11,12-methylene octadecanoic acid. PAO1, P. aeruginosa wild-type strain.*

### OAR Homologs Do Not Play Major Roles in *P. aeruginosa* Fatty Acid Synthesis

PA4389 and PA4786 had OAR activity both *in vitro* and *in vivo*. To further investigate their functions in *P. aeruginosa* fatty acid synthesis, the fatty acid compositions of the ΔPA4389 and ΔPA4786 mutant strains grown in LB medium were determined with gas chromatography–mass spectrometry (GC–MS). The fatty acid profiles of both mutant strains were almost the same as that of the wild-type strain PAO1 (Table 2). A comparison of the wild-type and OAR mutant strains with Tukey’s multiple-comparison test showed that the content of individual fatty acid species was not significantly altered in the *P. aeruginosa* strains (*P* > 0.05). This suggests that both ΔPA4389 and ΔPA4786 do not function predominantly in fatty acid synthesis under the conditions tested here. To confirm this, we first tested whether the expression of both genes affected their functions in fatty acid synthesis. We determined the expression level of PA4389 and PA4786 with qRT–PCR in PAO1 grown in LB. Under the test conditions, both PA4389 and PA4786 were expressed well, although the expression level of PA4389 was two–threefold higher than that of PA2967 (*fabG*), and the expression of PA4786 was only 30–40% of that of PA2967. This indicates that the lack of difference in the fatty acid compositions of the mutant strain and PAO1 is not attributable to the poor expression of these genes. Next, we attempted to delete PA2967 (*fabG*) when PA4389 or PA4786 was overexpressed from a plasmid-encoded gene in PAO1. However, although several independent experiments were performed, no PA2967-deleted strain was isolated, which implies that neither PA4389 nor PA4786 can replace the function of PA2967 (*fabG*) in PAO1.

We also tested the fatty acid profiles of the remaining OAR-homolog-deleted mutants. We found that the fatty acid compositions of none of these mutants differed significantly from that of the wild-type strain (data not shown).

### Mutation of Some OAR Genes Affects the Production of QS Signals in *P. aeruginosa*

In *P. aeruginosa*, the fatty acid synthetic reaction supplies many precursors for the production of many primary and secondary metabolic exoproducts, including three QS signals, the siderophore pyoverdine, and rhamnolipids (Zhu and Rock, 2008; Hannauer et al., 2012; Jimenez et al., 2012). To test whether these OAR homologs specifically synthesize these exoproducts, the production of some exoproducts was examined in the *P. aeruginosa* mutant strains.

**FIGURE 2:**
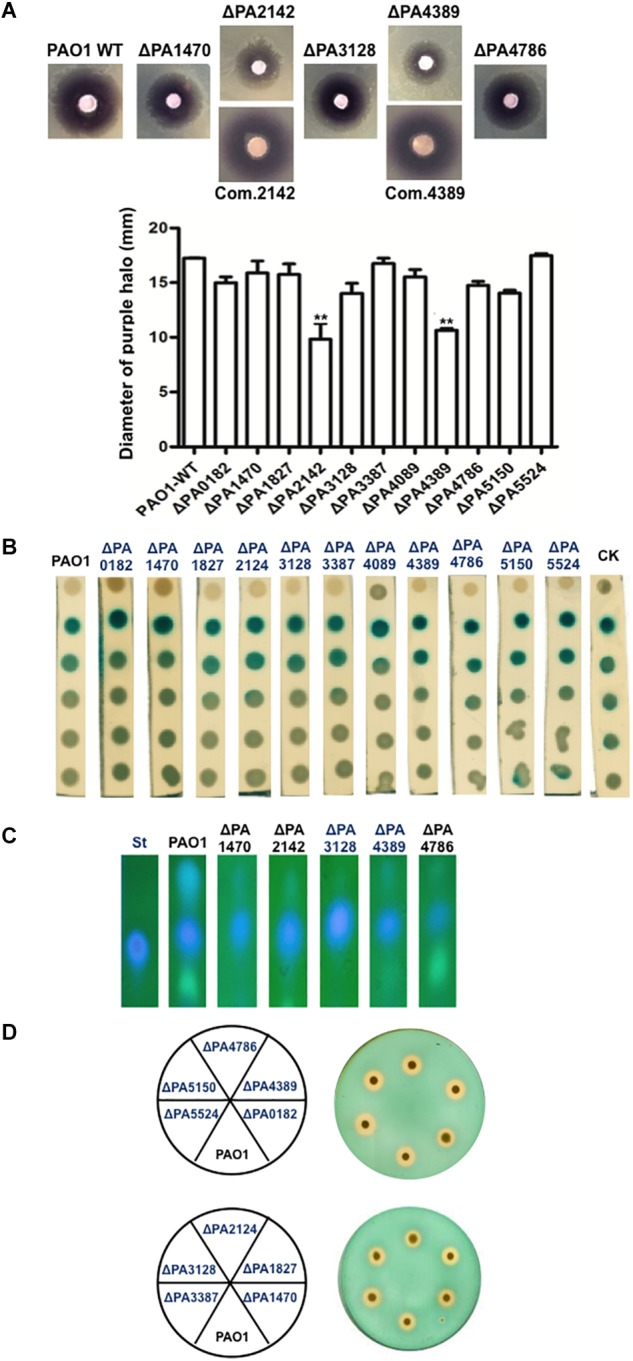
Detection of quorum-sensing (QS) signals and siderophore produced by *P. aeruginosa* strains. **(A)** Analysis of the C4-HSL signals produced by *P. aeruginosa* strains using *Chromobacterium violaceum* CV026 as reporter strain. **(B)** Analysis of the 3-oxo-C12-HSL signals produced by *P. aeruginosa* strains using *Agrobacterium tumefaciens* reporter strain NTL4 (pZLR4). **(C)** The amount of PQS produced by *P. aeruginosa* strains was compared by direct UV illumination of culture supernatant extracts separated by normal-phase TLC on silica 60 *F*_254_ plates. **(D)** Siderophore secretion in *P. aeruginosa* mutant strains. At least three independent repetitions of each experiment were performed. Black bars represent standard deviations. Pairwise comparisons were made between wild-type strain PAO1 and the OAR mutant strains with Student’s *t*-test. ^∗∗^Significant difference, *P* < 0.05. PAO1, *P. aeruginosa* wild type strain; ΔPAxxxx, *P. aeruginosa* OAR mutant strain; Com.xxxx, ΔPAxxxx strain carrying a plasmid-encoded PAxxxx gene. St, Standard PQS. CK, *A. tumefaciens* NT1/pTiC58Δ*accR*.

The QS signals, including *N*-butanoyl-L-homoserine lactone (C_4_-HSL), N-3-oxododecanoyl-L-homoserine lactone (3-oxo-C_12_-HSL), and PQS were extracted from the culture supernatants of the *P. aeruginosa* strains. The C_4_-HSL signal was detected with an agar overlay of the *C. violaceum* reporter strain CV026, which produces a purple halo in response to acyl-HSLs. The purple halos around the ΔPA2142 and ΔPA4389 mutant strains were much weaker than that around wild-type strain PAO1, and the diameters of purple halos of these two mutant strains were significantly smaller than that of strain PAO1 (*P* < 0.01). However, complementation of both mutant strains with a plasmid encoding the corresponding wild-type OAR gene restored the high-level production of the C_4_-HSL signal (Figure 2A). The halos produced by the remaining OAR-homolog mutants did not differ markedly from that of the wild-type strain (Figure 2A) (*P* > 0.05). The 3-oxo-C_12_-HSL signal was investigated using the reporter strain *A. tumefaciens* NL4 (pZLR4). The data showed that the amounts of 3-oxo-C12-HSL produced by all *P. aeruginosa* mutant strains were almost same as that produced by the wild type strain PAO1 (Figure 2B). We also examined the PQS signal produced by the *P. aeruginosa* mutant strains using thin-layer chromatography (TLC). The level of PQS was reduced by 50–60% in the supernatant extract of ΔPA1470, ΔPA4389, and ΔPA4786, but was increased by about 20% in the supernatant extract of ΔPA3128 (Figure 2C). The amounts of PQS in the remaining mutant strains did not differ markedly from that of wild-type strain PAO1 (data not shown). From these data, we infer that PA4389 and PA4786 are involved in the production of QS signals, even though neither enzyme functions in the fatty acid synthetic pathway. Although neither protein showed OAR activity, PA1470, PA2142, and PA3128 affected the ability of *P. aeruginosa* to produce QS signals.

The secretion of pyoverdine by the mutant *P. aeruginosa* strains was examined by culturing them on LB-chrome azurol S (CAS) indicator plates. All the mutant strains generated prominent clear zones similar to that generated by the wild-type strain PAO1 (Figure 2D). The production of rhamnolipids by the mutant *P. aeruginosa* strains was also detected, but the deletion of the OAR homolog gene did not affect the ability of *P. aeruginosa* to secrete rhamnolipids (data not shown). These data suggest that none of the OAR homologs are involved in the secretion of pyoverdine or rhamnolipids.

### Deletion of Some OAR Genes Affected *P. aeruginosa* Swarming Motility and Biofilm Formation

**FIGURE 3:**
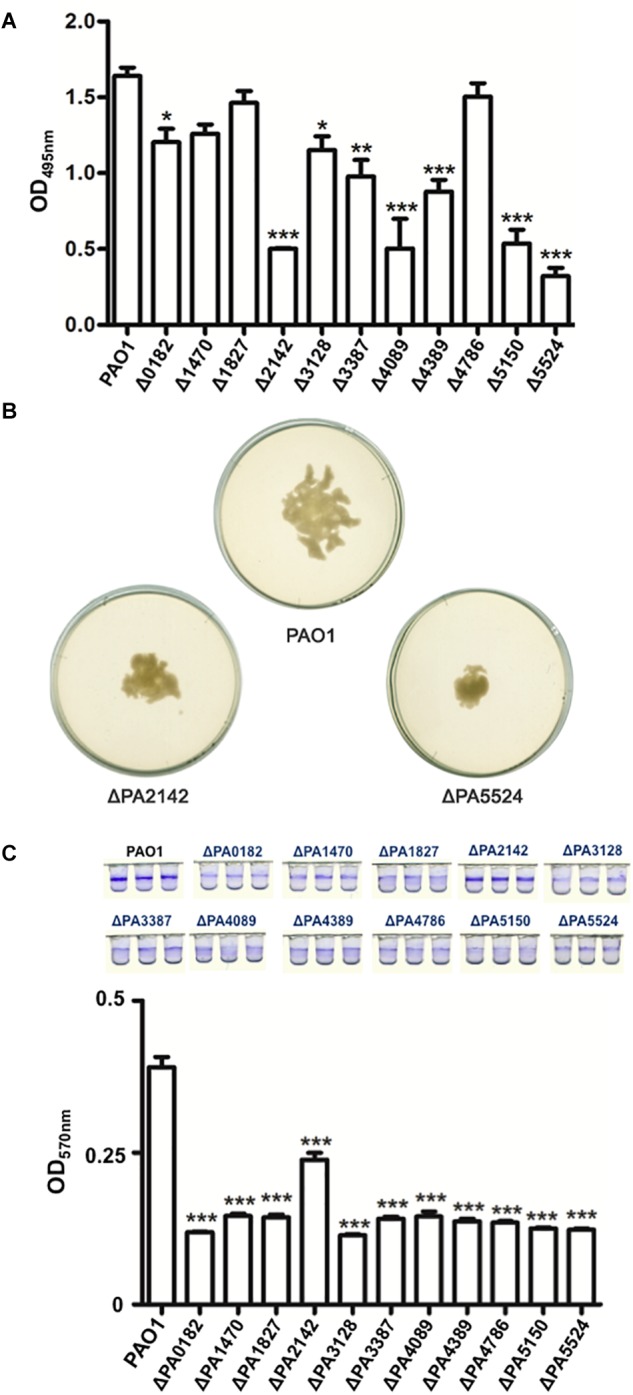
Analysis of LasB activities, swarming motility and biofilm formation of *P. aeruginosa* strains. **(A)** LasB protease activities in culture supernatants of *P. aeruginosa* strains were measured using a Congo red-elastin dye release assay. Data are the mean ± standard deviation of triplicate measurements. Pair-wise comparisons were made between wild type strain PAO1 and OAR mutant strains by Student’s *t*-test. ^∗^Significant difference, *P* < 0.05. **(B)** Assay of swarming motility of *P. aeruginosa* strains on 0.5% agar plate. **(C)** Analysis of biofilm formation by *P. aeruginosa* strains using crystal violet stain. The amount of biomass in the biofilm was measured indirectly with spectrometry at 595 nm. Three independent repetitions were performed. Black bars represent standard deviations. Pairwise comparisons were made between wild-type strain PAO1 and the OAR mutant strains with Student’s *t*-test. ^∗^Significant difference, *P* < 0.05; ^∗∗^Significant difference, *P* < 0.05; ^∗∗∗^Significant difference, *P* < 0.01. PAO1, *P. aeruginosa* wild type strain; ΔPAxxxx, *P. aeruginosa* OAR mutant strain.

*Pseudomonas aeruginosa* is an important human pathogen (Moore and Flaws, 2011). Therefore, we next examined the effects of deleting these OAR genes on the virulence of *P. aeruginosa*. We first tested the production of LasA, LasB, and procyanin by the *P. aeruginosa* mutants. LasA protease activity was determined by measuring the ability of the *P. aeruginosa* culture supernatants to lyse boiled *S. aureus* cells. The LasA activity was not significantly altered in any mutant strain (*P* > 0.05) (data not shown). The elastase proteolytic activity (LasB) was measured in the *P. aeruginosa* mutants using the elastin Congo red assay. The LasB activity in the mutant strains varied relative to that in wild-type strain PAO1. The LasB activity was not significantly reduced in mutant strain ΔPA1470, ΔPA1827, or ΔPA4786 (*P* > 0.5), whereas the LasB activity in strains ΔPA0182, ΔPA3128, ΔPA3387, and ΔPA4389 was reduced by 30–40% (*P* < 0.05), and in ΔPA2142, ΔPA4089, ΔPA5150, and ΔPA5524, the LasB activity was only one third of that in strain PAO1 (*P* < 0.01) (Figure 3A). The production of procyanin by the OAR homolog mutants was also determined, but none of the OAR homolog gene mutations affected the ability of *P. aeruginosa* to produce procyanin (data not shown).

We then tested the swarming motility of the OAR homolog mutants on semisolid plates (containing 0.5% agar) at 30°C. Of the 11 OAR homolog genes tested, only the deletion of PA2142 or PA5524 reduced the swarming motility of *P. aeruginosa*, whereas the mutation of the remaining genes had no effect (Figure 3B). We also examined the biofilm formation of these mutant strains, and the deletion of the OAR genes significantly reduced biofilm formation (*P* < 0.01) (Figure 3C).

### Some OAR Homologs Affect the Utilization of Specific Carbon Sources

**FIGURE 4:**
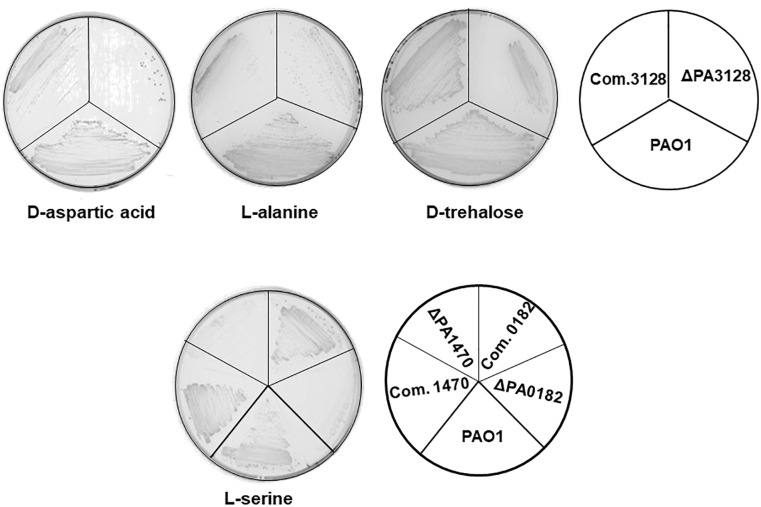
Growth of *P. aeruginosa* strains on ME medium supplemented with specific chemical compounds as the carbon source. Chemical compounds included D-aspartic acid, L-alanine, L-serine, and D-trehalose at final concentrations of 100 mmol/L. PAO1, *P. aeruginosa* wild-type strain; ΔPAxxxx, *P. aeruginosa* OAR mutant strain. Com.xxxx, ΔPAxxxx strain carrying a plasmid-encoded PAxxxx gene.

*Pseudomonas aeruginosa* cells are capable of metabolizing 70–80 different organic substrates as sole carbon sources for growth, including sugars, sugar alcohols, amino acids, and organic acids (Stanier et al., 1966). Its capacity to utilize a wide spectrum of substrates probably contributes to the survival and proliferation of *P. aeruginosa* in nutritionally complex environments and animal hosts. The SDR superfamily of proteins is involved in the oxidation–reduction reactions of many catabolic pathways (Kallberg et al., 2002; Oppermann et al., 2003). Therefore, to investigate whether the *P. aeruginosa* OAR homologs are involved in the utilization of specific carbon sources to support growth, we first tested the growth of the OAR homolog mutants in mineral medium containing 68 different substrates as the sole carbon source (Supplementary Table S4) using Biolog Gen III MicroPlates^TM^. The results confirmed that several OAR homologs are involved in the metabolism of specific carbon sources. As shown in Table 3, six OAR genes seemed to be required for *P. aeruginosa* growth on specific carbon sources. The ΔPA3128 mutant grew as well as the wild-type strain PAO1 in rich medium, but its growth was weaker than that of the wild-type strain in mineral medium supplemented with many different carbon sources, including sugars, sugar alcohols, organic acids, and amino acids. Three mutant strains, ΔPA2142, ΔPA4389, and ΔPA4786, grew more slowly than the wild-type strain PAO1 in mineral medium supplemented with *N*-acetyl-β-D-mannosamine, and two OAR genes, PA0182 and PA1470, were required for the utilization of several amino acids, including L-serine, L-aspartic acid, L-glutamic acid, and L-histidine (Table 3). To confirm this observation, we tested the growth of these OAR mutants on ME medium supplemented with specific carbon sources. ΔPA3128 failed to grow on ME supplemented with D-aspartic acid and grew more weakly than wild-type strain PAO1 on ME supplemented with L-alanine or D-trehalose (Figure 4). In liquid medium, the final cell densities of ΔPA3128 were only 20–40% of those of the wild-type strain. The overall cell yields of mutant ΔPA1470 and ΔPA0182 on ME supplemented with L-serine were also significantly lower (<70%) than those of wild-type strain PAO1 (Figure 4). We also complemented these mutants with a plasmid encoding the corresponding wild-type OAR gene, and found that all these OAR genes restored the growth of the corresponding mutant on ME supplemented with specific carbon sources (Figure 4). However, unlike their growth in a 96-well plate, mutants ΔPA2142, ΔPA4389, and ΔPA4786, grew as well as wild-type strain PAO1 on ME medium supplemented with *N*-acetyl-β-D-mannosamine.

The remaining five OAR genes did not seem to be involved in the utilization of carbon sources (Table 3). Based on the growth patterns of the mutants on different sole carbon sources, the deletion of PA1827, PA3387, PA4089, PA5150, or PA5524 did not affect the ability of *P. aeruginosa* to metabolize these substrates, and all these mutants grew as well as the wild-type strain PAO1 on these different sole carbon sources.

**Table 3:** Carbon utilization by *P. aeruginosa* OAR-homolog-mutant strains.

Mutant ORF	Carbon utilization deficiency
PA0182	Inosine, α-D-glucose, L-aspartic acid, L-glutamic, L-histidine, L-pyroglutamic acid, L-serine, D-gluconic acid, α-hydroxy-butyric acid
PA1470	L-aspartic acid, L-glutamic acid, L-histidine, L-pyroglutamic acid
PA1827	None
PA2142	*N*-acetyl-β-D-mannosamine
PA3128	D-Trehalose, D-turanose, α-D-glucose, D-fructose, D-arabitol, α-hydroxy-butyric acid, glycerol, gelatin, glycyl-L-prolin, L-alanine, L-arginine, L-aspartic acid, L-glutamic acid, L-histidine, L-pyroglutamic acid, L-serine, quinic acid
PA3387	None
PA4089	None
PA4389	D-turanose, *N*-acetyl-β-D-mannosamine, L-serine
PA4786	*N*-acetyl-β-D-mannosamine, gelatin
PA5150	None
PA5524	None

## Discussion

In this study, we characterized 12 OAR homologs of *P. aeruginosa*. A bioinformatic analysis showed that all *P. aeruginosa* OAR homologs have a similar structure and conserved catalytic triad to those of the bacterial OAR enzymes. However, experimental studies showed that only the proteins encoded by PA2967, PA4389, and PA4786 had OAR activity *in vivo* and *in vitro*, which suggests that not all *P. aeruginosa* OAR homologs can be designated OAR enzymes. PA2967, located in the fatty acid synthetic gene cluster, encodes FabG, which has been identified as a key enzyme in fatty acid synthesis in *P. aeruginosa* (Kutchma et al., 1999; Hoang et al., 2002). Therefore, the function of FabG is not discussed in this paper.

The proteins encoded by PA4389 or PA4786 also displayed OAR activity, but the deletion of either gene did not significantly alter the fatty acid composition of the mutant strains. Neither of these two genes restored the function of PA2967 in *P. aeruginosa*, which implies that neither protein functions predominantly in the fatty acid synthetic pathway.

Our data show that the production of the C_4_-HSL QS signal was reduced in the ΔPA4389 mutant strain and that the PQS QS signal in the ΔPA4786 and ΔPA4389 strains was only 40–50% of that in wild-type strain PAO1. Both the C_4_-HSL and PQS signals are fatty-acid derivatives, which are converted from precursors produced in the fatty acid biosynthetic pathway. Although PA4389 or PA4786 do not play major roles in fatty acid synthesis in *P. aeruginosa*, because both proteins have OAR activity, the deletion of either of these two genes might reduce the production of some specific intermediates used to synthesize QS signals, thus reducing QS signal production. It has been reported that in *X. campestris* pv. *campestris*, the reduction of the total cellular OAR activities reduces the production of diffusible signal factor (DSF) (Deng et al., 2015; Hu et al., 2018). Therefore, the reduction in the C_4_-HSL or PQS signal might be related to the reduced total cellular OAR activity in the mutant strains.

The SDR superfamily proteins are involved in the oxidation–reduction reactions of many catabolic pathways (Kallberg et al., 2002; Oppermann et al., 2003), and OAR proteins belong to the SDR superfamily (Wang and Cronan, 2004). Therefore, some OAR proteins that do not have OAR activity or function in the fatty acid synthetic pathway may be involved in the metabolic pathways of other substances. Our study of the carbon source utilization of the *P. aeruginosa* mutants confirmed this view. The products of nine OAR genes had no OAR activity, and three of these genes, PA0182, PA1470, and PA3128, may be involved in the utilization of specific carbon sources. The ΔPA3128 mutant failed to use D-aspartic acid as a carbon source and was defective in the metabolism of L-alanine and D-trehalose. The PA3128 gene is located alone on the bacterial chromosome, and its product shares 60% identical residues with SMc02486, which is considered to play a role in the tricarboxylic acid (TCA) cycle of *S. meliloti* (Jacob et al., 2008). Therefore, the growth phenotype of the ΔPA3128 mutant may be attributable to defects in the TCA cycle. The growth of the ΔPA1470 and ΔPA0182 mutants was weaker than that of wild-type strain PAO1 in M9 supplemented with L-serine, suggesting that the OAR genes PA0182 and PA1470 are required for the utilization of L-serine, but this requires experimental confirmation.

The remaining OAR homologs, PA1827, PA2142, PA3387, PA4089, PA5150, and PA5524, did not affect the metabolism of carbon substrates by *P. aeruginosa*. The deletion of PA1827 did not affect the *P. aeruginosa* phenotype, except that its biofilm formation was reduced. The deletion of PA2142 produced pleiotropic effects, including reduced C_4_-HSL production, reduced LasB activity, and attenuated swarming motility in the mutant strain. PA3387 encodes RhlG, which has been well studied, and our data did not attribute any new functions to the product of this gene. Both PA4089 and PA5524 are located in a gene cluster involved in amino acid metabolism, whereas PA5150 occurs adjacent to a gene cluster encoding an ABC transporter system. However, they do not seem to function in amino acid metabolism. However, the deletion of one of these genes reduced LasB activity and biofilm formation in *P. aeruginosa*. The mutation of PA5524 also attenuated the swarming motility of *P. aeruginosa*. However, the detailed mechanisms underlying these effects require further investigation.

## Author Contributions

Q-QG and CZ cloned the OAR genes and constructed OAR deleted mutants. Q-QG tested the production of QS signals and motility of mutant strains, and carried out biochemical studies. W-BZ purified OAR proteins and tested the activity of OAR *in vitro*. Y-LS and Y-LL analyzed fatty acids composition of OAR mutant strains. Y-HY carried out experiments on the pathogenesis of *P. aeruginosa* strains. J-CM participated in the design of the study and helped to draft the manuscript. H-HW conceived of the study, and participated in its design and coordination, and helped to draft the manuscript. All authors read and approved the final manuscript.

## Conflict of Interest Statement

The authors declare that the research was conducted in the absence of any commercial or financial relationships that could be construed as a potential conflict of interest.
