# Supplementary material for: Characterization of 3-Oxacyl-Acyl Carrier Protein Reductase Homolog Genes in Pseudomonas aeruginosa PAO1
Source: Front Microbiol. 2019 May 22;10:1028. doi: 10.3389/fmicb.2019.01028 (PMC6558427; doi:10.3389/fmicb.2019.01028)
Supplement: Supplementary file 1 [file Table_1.DOCX]

**Characterization of 3-oxacyl-acyl carrier protein reductase homologue genes in *Pseudomonas aeruginosa* PAO1**

Qiao-Qiao Guo^1^, Wen-Bin Zhang^1^, Chao Zhang^1^, Yu-Lu Song^1^, Jin-Cheng Ma^1^, Yu-Ling Liao^1^, Yong-Hong Yu^2^ and Hai-Hong Wang^1^*

Supplementary Tables

**Table S1. Strains and plasmids used in this work.**

| **Strains and plasmids** | **Relevant Characteristics*^a^*** | **Source** |
| --- | --- | --- |
| **Strains** |  |  |
| ***E. coli*** | | |
| **DH5α** | F*^-^ deoR* *endA1 hsdR17 recA1*  ∆(l*acZYA*-*argF*) U169 (ϕ80*lacZ∆M15*) | TaKaRa |
| **Top 10** | F^-^∆ (*lac*)X74*deoRrecA1araD139*∆(*ara-leu*)*7697endA1*(ϕ80*lacZ*del*M15*) | TaKaRa |
| **BL21 (DE3)** | *E.coli* B; F^-^ *ompT* r_B_^-^m_B_^-^ (λDE3) | TaKaRa |
| **S17-1** | F- *thi* *pro* *hsdR* [RP4-2 Tc::Mu Km::Tn7 (Tp Sm)] | CGSC |
| **CL104** | *fabG* (Ts) *panD* Cm^r^, Tet^r^, Km^r^ | (17) |
| ***P. aeruginosa*** | | |
| **PAO1** | Wild type | ATCC |
| **ΔPA0182** | Deletion of PA0182 in PAO1 | This study |
| **ΔPA1470** | Deletion of PA1470 in PAO1 | This study |
| **ΔPA1827** | Deletion of PA1827 in PAO1 | This study |
| **ΔPA2142** | Deletion of PA2142 in PAO1 | This study |
| **ΔPA3128** | Deletion of PA3128 in PAO1 | This study |
| **ΔPA3387** | Deletion of PA3387 in PAO1 | This study |
| **ΔPA4089** | Deletion of PA4089 in PAO1 | This study |
| **ΔPA4389** | Deletion of PA4389 in PAO1 | This study |
| **ΔPA4786** | Deletion of PA4786 in PAO1 | This study |
| **ΔPA5150** | Deletion of PA5150 in PAO1 | This study |
| **ΔPA5524** | Deletion of PA5524 in PAO1 | This study |
| **Plasmids** |  |  |
| **pBAD24M** | Amp^r^; NcoI site of pBAD24 replaced by NdeI site | (40) |
| **pBAD24M-EcG** | Amp^r^, pBAD24M carrying *E. coli* *fabG* | This study |
| **pMD19** | Amp^r^, T-vector | Takara |
| **pET28 (b)** | Km^r^, expression vector | Novagen |
| **pK18mobsacB** | Km^r^; *sacB*-based gene replacement vector | (30) |
| **pMD19-0182** | Amp^r^, 750 bp PCR DNA fragment of PA0182 inserted into pMD19 | This study |
| **pMD19-1470** | Amp^r^, 750 bp PCR DNA fragment of PA1470 inserted into pMD19 | This study |
| **pMD19-1827** | Amp^r^, 750 bp PCR DNA fragment of PA1827 inserted into pMD19 | This study |
| **pMD19-2142** | Amp^r^, 750 bp PCR DNA fragment of PA2142 inserted into pMD19 | This study |
| **pMD19-2967** | Amp^r^, 750 bp PCR DNA fragment of PA2967 inserted into pMD19 | This study |
| **pMD19-3128** | Amp^r^, 750 bp PCR DNA fragment of PA3128 inserted into pMD19 | This study |
| **pMD19-3387** | Amp^r^, 750 bp PCR DNA fragment of PA3387 inserted into pMD19 | This study |
| **pMD19-4089** | Amp^r^, 750 bp PCR DNA fragment of PA4089 inserted into pMD19 | This study |
| **pMD19-4389** | Amp^r^, 750 bp PCR DNA fragment of PA4389 inserted into pMD19 | This study |
| **pMD19-4786** | Amp^r^, 1350 bp PCR DNA fragment of PA4786 inserted into pMD19 | This study |
| **pMD19-5150** | Amp^r^, 750 bp PCR DNA fragment of PA5150 inserted into pMD19 | This study |
| **pMD19-5524** | Amp^r^, 750 bp PCR DNA fragment of PA5524 inserted into pMD19 | This study |
| **pBAD24M-PA0182** | Amp^r^, the NdeI-HindIII fragment of pMD19-0182 inserted into same sites of pBAD24M | This study |
| **pBAD24M-PA1470** | Amp^r^, the NdeI-HindIII fragment of pMD19-1470 inserted into same sites of pBAD24M | This study |
| **pBAD24M-PA1827** | Amp^r^, the NdeI-HindIII fragment of pMD19-1827 inserted into same sites of pBAD24M | This study |
| **pBAD24M-PA2142** | Amp^r^, the NdeI-HindIII fragment of pMD19-2142 inserted into same sites of pBAD24M | This study |
| **pBAD24M-PA2967** | Amp^r^, the NdeI-HindIII fragment of pMD19-12967 inserted into same sites of pBAD24M | This study |
| **pBAD24M-PA3128** | Amp^r^, the NdeI-HindIII fragment of pMD19-3128 inserted into same sites of pBAD24M | This study |
| **pBAD24M-PA3387** | Amp^r^, the NdeI-HindIII fragment of pMD19-3387 inserted into same sites of pBAD24M | This study |
| **pBAD24M-PA4089** | Amp^r^, the NdeI-HindIII fragment of pMD19-4089 inserted into same sites of pBAD24M | This study |
| **pBAD24M-PA4389** | Amp^r^, the NdeI-HindIII fragment of pMD19-4389 inserted into same sites of pBAD24M | This study |
| **pBAD24M-PA4786** | Amp^r^, the NdeI-HindIII fragment of pMD19-4786 inserted into same sites of pBAD24M | This study |
| **pBAD24M-PA5150** | Amp^r^, the NdeI-SalI fragment of pMD19-5150 inserted into same sites of pBAD24M | This study |
| **pBAD24M-PA5524** | Amp^r^, the NdeI-HindIII fragment of pMD19-5524 inserted into same sites of pBAD24M | This study |
| **pET-28b-PA0182** | Km^r^ , PA0182 in pET-28b. | This study |
| **pET-28b-PA1470** | Km^r^ , PA1470 in pET-28b. | This study |
| **pET-28b-PA1827** | Km^r^ , PA1827 in pET-28b. | This study |
| **pET-28b-PA2142** | Km^r^ , PA2142 in pET-28b. | This study |
| **pET-28b-PA2967** | Km^r^ , PA2967 in pET-28b. | This study |
| **pET-28b-PA3128** | Km^r^ , PA3128 in pET-28b. | This study |
| **pET-28b-PA3387** | Km^r^ , PA3387 in pET-28b. | This study |
| **pET-28b-PA4089** | Km^r^ , PA4089 in pET-28b. | This study |
| **pET-28b-PA4389** | Km^r^ , PA4389 in pET-28b. | This study |
| **pET-28b-PA4786** | Km^r^ , PA4786 in pET-28b. | This study |
| **pET-28b-PA5150** | Km^r^ , PA5150 in pET-28b. | This study |
| **pET-28b-PA5524** | Km^r^ , PA5524 in pET-28b. | This study |
| **pK18-PA0182** | Gm^r^ , a 1,200 bp DNA fragment containing Up PA0182 and Down PA0182 inserted into pK18mobsacB between BamHI and HindIII sites. | This study |
| **pK18-PA1470** | Gm^r^ , a 1,200 bp DNA fragment containing Up PA1470 and Down PA1470 inserted into pK18mobsacB between EcoRI and HindIII sites. | This study |
| **pK18-PA1827** | Gm^r^ , a 1,200 bp DNA fragment containing Up PA1827 and Down PA1827 inserted into pK18mobsacB between EcoRI and HindIII sites. | This study |
| **pK18-PA2142** | Gm^r^ , a 1,200 bp DNA fragment containing Up PA2142 and Down PA2142 inserted into pK18mobsacB between BamHI and PstI sites. | This study |
| **pK18-PA2967** | Gm^r^ , a 1,200 bp DNA fragment containing Up PA2967 and Down PA2967 inserted into pK18mobsacB between BamHI and HindIII sites. | This study |
| **pK18-PA3128** | Gm^r^ , a 1,200 bp DNA fragment containing Up PA3128 and Down PA3128 inserted into pK18mobsacB between EcoRI and HindIII sites. | This study |
| **pK18-PA3387** | Gm^r^ , a 1,200 bp DNA fragment containing Up PA3387 and Down PA3387 inserted into pK18mobsacB between BamHI and HindIII sites. | This study |
| **pK18-PA4089** | Gm^r^ , a 1,200 bp DNA fragment containing Up PA4089 and Down PA4089 inserted into pK18mobsacB between EcoRI and HindIII sites. | This study |
| **pK18-PA4389** | Gm^r^ , a 1,200 bp DNA fragment containing Up PA4389 and Down PA4389 inserted into pK18mobsacB between EcoRI and HindIII sites. | This study |
| **pK18-PA4786** | Gm^r^ , a 1,200 bp DNA fragment containing Up PA4786 and Down PA4786 inserted into pK18mobsacB between EcoRI and HindIII sites. | This study |
| **pK18-PA5150** | Gm^r^ , a 1,200 bp DNA fragment containing Up PA5150 and Down PA5150 inserted into pK18mobsacB between EcoRI and BamHI sites. | This study |
| **pK18-PA5524** | Gm^r^ , a 1,200 bp DNA fragment containing Up PA5524 and Down PA5524 inserted into pK18mobsacB between BamHI and HindIII sites. | This study |

*^a^* Gm, Km and Amp denote gentamicin, kanamycin and ampicillin，respectively.

**Table S2. Sequences of the PCR primers used in this work**

| Primer name | Primer sequence (5’ to 3’) | Digestion sites ^a^ |
| --- | --- | --- |
| PA0182F-NdeI | GCGCCATATGTCCGTATCCTCTGCATC | NdeI |
| PA0182R-HindIII | GCGCAAGCTTCAGGCCGAGAAACCGCC | HindIII |
| PA1479F-NdeI | GCGCCATATGCCTGACATCACCCAAG | NdeI |
| PA1479R-HindIII | GCGCAAGCTTCAGGCGAAACCGCCGTT | HindIII |
| PA1827F-NdeI | GCGCCATATGCACACTCATTCTCCAAT | NdeI |
| PA1827R-HindIII | GCGCAAGCTTCAGTTGCCGACCCGCC | HindIII |
| PA2142F-NdeI | GCGCCATATGAGCGAACAGCGACAGA | NdeI |
| PA2142R-HindIII | GCGCAAGCTTCAGCCGTTGACTACCGTA | HindIII |
| PA2967F-NdeI | GCGCCATATGAGTCTGCAAGGTAAGG | NdeI |
| PA2967R-HindIII | GCGCAAGCTTCAGCTCATGTACATCCCA | HindIII |
| PA3128F-NdeI | GCGCCATATGCGCAACGTCATGCTGAT | NdeI |
| PA3128R-HindIII | GCGCAAGCTTCTAGCGGCCGCCGCTGA | HindIII |
| PA3387F-NdeI | GCGCCATATGCATCCCTATTTCAGTCT | NdeI |
| PA3387R-HindIII | GCGCAAGCTTCAGAGATGAAAACCGCC | HindIII |
| PA4089F-NdeI | GCGCCATATGAGGCGCTTGGAGAACCG | NdeI |
| PA4089R-HindIII | GCGCAAGCTTCAGGCCAGCCCGCCGTTTT | HindIII |
| PA4389-NdeI | GCGCCATATGCAATTGAAAGACAAGGT | NdeI |
| PA4389R-HindIII | GCGCAAGCTTCTACAGGCGCAGGCCG | HindIII |
| PA4786F-NdeI | GCGCCATATGACCGATCGTTACATCGC | NdeI |
| PA4786R-HindIII | GCGCAAGCTTCAGGCCCCCAGCAGGC | HindIII |
| PA5150F-NdeI | GCGCCATATGGCGGAGAGAAAGACCCT | NdeI |
| PA5150R-SalI | GCGCGTCGACACGGCGGGCAGCACGTCTA | SalI |
| PA5524F-NdeI | GCGCCATATGAATGACTTTTCGAAGTGG | NdeI |
| PA5524R-HindIII | GCGCAAGCTTCGAAAACAAACGGAGCTGA | HindIII |
| PA0182 up1 BamHI | CGCGGATCCAGTTCGAAACCGCCGCCGAT | BamHI |
| PA0182 dn1 | GGAAATCTCCATCGGAGTGG |  |
| PA0182 up2 | TCCACTCCGATGGAGATTTCCCCCGGGTCGCCTCGTTC |  |
| PA0182 dn2 HindIII | CCCAAGCTTCATGCCTTCGCCACGGTGAT | HindIII |
| PA1470 up1 EcoRI | CCGGAATTCACATCCGGGTCGCCATCGGT | EcoRI |
| PA1470 dn1 | CTGGGACTCTCCTGGAATGA |  |
| PA1470 up2 | TCATTCCAGGAGAGTCCCAGCGCCGTGTGGGCGGGGCACGA |  |
| PA1470 dn2 HindIII | CCCAAGCTTTTCCGGAGAAGTCTCCGACT | HindIII |
| PA1827 up1 EcoRI | CCGGAATTCATCAGGCCGGCATCGATGC | EcoRI |
| PA1827 dn1 | GACGGGCTTCCTCTGTAATG |  |
| PA1827 up2 | CATTACAGAGGAAGCCCGTCAGTGGAAGCGGCGGGTAAC |  |
| PA1827 dn2 HindIII | CCCAAGCTTCGGCAAGCTGATGAAGGAAC | HindIII |
| PA2142 up1 BamHI | CGCGGATCCGCGTTGAAGGAGACCTTTCC | BamHI |
| PA2142 dn1 | GGTGGTTTCCTCTCTGTCGG |  |
| PA2142 up2 | TCCGACAGAGAGGAAACCACCGGCGCCCCGGCGACCGCGTT |  |
| PA2142 dn2 PstI | AACTGCAGGATCAACCCCCAGTCGCGTT | PstI |
| PA2967 up1 BamHI | CGCGGATCCTGATGCAGCAGGCGGTTCC | BamHI |
| PA2967 dn1 | GGGATTCTCTCCTTTCTCTC |  |
| PA2967 up2 | GAGAGAAAGGAGAGAATCCCATGTGACGTTACCTTTCTCG |  |
| PA2967 dn2 HindIII | CCCAAGCTTGACTACCAGAGTAGGTAAGG | HindIII |
| PA3128 up1 BamHI | CGCGGATCCATTCCACGCATCACGGCCCT | BamHI |
| PA3128 dn1 | GATGATGCTCCCCAGGTCAG |  |
| PA3128 up2 | CTGACCTGGGGAGCATCATCGCCTGGTACGCAAGCTAGGC |  |
| PA3128 dn2 HindIII | CCCAAGCTTATTGCGGTGACCTCGAAGAT | HindIII |
| PA3387 up1 EcoRI | CCGGAATTCATGGCCAAACCATGGGCGA | EcoRI |
| PA3387 dn1 | GGGAATGACTCCGGGCTGG |  |
| PA3387 up2 | CCCAGCCCGGAGTCATTCCCGCGCCGCCAATCCGGGTAA |  |
| PA3387 dn2 HindIII | CCCAAGCTTAACCGCTGGCCGTGCTCAT | HindIII |
| PA4089 up1 EcoRI | CCGGAATTCACTTCTTCGTCCTCGGCAA | EcoRI |
| PA4089 dn1 | GATGGGCGCTCCAGCAGTT |  |
| PA4089 up2 | GAACTGCTGGAGCGCCCATCAGCCGCTGGCGTTCGCCCT |  |
| PA4089 dn2 HindIII | CCCAAGCTTTGCCAGGCAATCTCCAGGC | HindIII |
| PA4389 up1 EcoRI | CCGGAATTCCAGCACCCAGAAGTCGAA | EcoRI |
| PA4389 dn1 | TGCTTGGAACCCCACGAGCA |  |
| PA4389 up2 | TGCTCGTGGGGTTCCAAGCAGCCCCGGCCCCGCAAGGAAA |  |
| PA4389 dn2 HindIII | CCCAAGCTTAACGGCCAGGTCAGCACC | HindIII |
| PA4786 up1 EcoRI | CCGGAATTCGGCGATCTTGTTGGCCAC | EcoRI |
| PA4786 dn1 | GGAACGGGCTCCCGGTGGAA |  |
| PA4786 up2 | TTCCACCGGGAGCCCGTTCCGGCGGCCCCTGACTCCC |  |
| PA4786 dn2 HindIII | CCCAAGCTTCGGCTGCCCGACGATCCT | HindIII |
| PA5150 up1 EcoRI | CCGGAATTCACACCACACCGGACTCAT | EcoRI |
| PA5150 dn1 | ACGCCCTCCGTGAAGGCCT |  |
| PA5150 up2 | CAGGCCTTCACGGAGGGCGTGCGAACGGCGGAAAGCAAA |  |
| PA5150 dn2 BamHI | CGCGGATCCACCACGGCAGCCTGCTCAA | BamHI |
| PA5524 up1 BamHI | CGCGGATCCAATACCAGCACCACCTTG | BamHI |
| PA5524 dn1 | TTTTGTGATCACAAAATTGACATGG |  |
| PA5524 up2 | TCAATTTTGTGATCACAAAAACATGTGCAACTGCAGCCTG |  |
| PA5524 dn2 HindIII | CCCAAGCTTGAACAGCCGGTTGAAGAAC | HindIII |

*^a^* underlined nucleotide sequences are digestion sites of restriction endonuclease.

**Table S3. Swiss-Modeling for *P. aeruginosa* OAR homologues**

| OAR homologues | *E.coli* FabG (PDB No. 1q7c) | | |
| --- | --- | --- | --- |
|  | **Seq Identity (%)** | **GMQE** | **QMEAN** |
| PA1827 | 40.83 | 0.73 | -1.15 |
| PA3387 | 34.16 | 0.68 | -3.49 |
| PA4089 | 38.33 | 0.73 | -1.38 |
| PA4389 | 37.08 | 0.72 | -2.3 |
| PA5150 | 34.63 | 0.70 | -2.23 |
| PA5524 | 42.68 | 0.72 | -1.38 |
|  | ***S. aureus* FabG1 (PDB No. 3sj7)** | | |
| PA1470 | 40.42 | 0.73 | -2.39 |
| PA3182 | 38.59 | 0.73 | -0.22 |
|  | ***P. aeruginosa* FabG(PDB No. 4bny)** | | |
| PA0182 | 31.19 | 0.69 | -1.899 |
|  | ***Synechocystis sp* FabG (PDB No. 4rzh)** | | |
| PA2142 | 40.59 | 0.63 | -1.74 |
|  | ***Mycobacterium tuberculosis* FabG4 (3v1t)** | | |
| PA4786 | 50.79 | 0.72 | -1.96 |

**Table S4. Carbon sources for OAR mutant strain screening**

| Pentoses and derivatives | α-Galactosides | Sugar alcohols | Amino acids and derivatives | Organic acids |
| --- | --- | --- | --- | --- |
| L-Rhamnose | D-Raffinose | D-Sorbitol | D-Aspartic Acid | p-Hydroxy-phenylacetic acid |
| Hexoses and derivatives | α-D-Lactose | D-Mannitol | D-Serine | L-Lactic acid |
| α-D-Glucose | D-Melibiose | D-Arabitol | Gelatin | α-Keto-glutaric acid |
| D-Mannose | **α-Glucosides** | Myo-inositol | Glycyl-L-prolin | D-Malic acid |
| D-Fructose | D-Maltose | Glycerol | L-Alanine | L-Malic acid |
| D-Galactose | D-Trehalose | **Sugar phosphates** | L-Arginine | Bromo-succinic acid |
| 3-Methyl glucose | Sucrose | D-Glucose-6-PO_4_ | L-Aspartic acid | γ-Amino-butryric acid |
| D-Fucose | D-Turanose | D-Fructose-6-PO_4_ | L-Glutamic acid | α-Hydroxy-butyric acid |
| L-Fucose |  |  | L-Histidine | Acetoacetic acid |
| β-Methyl-D-glucoside | **β-Glucosides** |  | L-Pyroglutamic acid | Propionic acid |
| N-Acetyl-D-glucosamine | D-Cellobiose | **Polysaccharide** | L-Serine | Acetic acid |
| N-Acetyl-β-D-mannosamine | Gentiobiose | Dextrin | N-Acetyl-neuraminic acid | Formic acid |
| N-Acetyl-D-galactosamine | D-Salicin | Stachyose |  | Mucic acid |
| D-Gluconic acid |  | Pectin | **Nucleotides and derivatives** | Quinic acid |
| D-Galacturonic |  |  | Inosine | D-Saccharic acid |
| L-Galactonic acid lactone |  | **Organic esters** |  | Citric acid |
|  |  | Methyl pyruvate |  |  |
| D-Glucuronic acid |  | D-Lactic acid methyl ester |  |  |
| Glucuronamide |  | Tween 40 |  |  |
